# Single Domain Antibodies Targeting Receptor Binding Pockets of NadA Restrain Adhesion of *Neisseria meningitidis* to Human Brain Microvascular Endothelial Cells

**DOI:** 10.3389/fmolb.2020.573281

**Published:** 2020-12-23

**Authors:** Amod Kulkarni, Evelína Mochnáčová, Petra Majerova, Ján Čurlík, Katarína Bhide, Patrícia Mertinková, Mangesh Bhide

**Affiliations:** ^1^Laboratory of Biomedical Microbiology and Immunology, The University of Veterinary Medicine and Pharmacy, Kosice, Slovakia; ^2^Institute of Neuroimmunology of Slovak Academy of Sciences, Bratislava, Slovakia

**Keywords:** *Neisseria*, HBMEC = human brain microvascular endothelial cell, phage display, VHH antibodies, adhesin, BBB–blood–brain barrier

## Abstract

*Neisseria* adhesin A (NadA), one of the surface adhesins of *Neisseria meningitides* (NM), interacts with several cell types including human brain microvascular endothelial cells (hBMECs) and play important role in the pathogenesis. Receptor binding pockets of NadA are localized on the globular head domain (A^33^ to K^69^) and the first coiled-coil domain (L^121^ to K^158^). Here, the phage display was used to develop a variable heavy chain domain (VHH) that can block receptor binding sites of recombinant NadA (rec-NadA). A phage library displaying VHH was panned against synthetic peptides (NadA-gd^A33−K69^ or NadA-cc^L121−K158^), gene encoding VHH was amplified from bound phages and re-cloned in the expression vector, and the soluble VHHs containing disulfide bonds were overexpressed in the SHuffle *E. coli*. From the repertoire of 96 clones, two VHHs (VHH_F3_–binding NadA-gd^A33−K69^ and VHH_G9_–binding NadA-cc^L121−K158^) were finally selected as they abrogated the interaction between rec-NadA and the cell receptor. Preincubation of NM with VHH_F3_ and VHH_G9_ significantly reduced the adhesion of NM on hBMECs *in situ* and hindered the traversal of NM across the *in-vitro* BBB model. The work presents a phage display pipeline with a single-round of panning to select receptor blocking VHHs. It also demonstrates the production of soluble and functional VHHs, which blocked the interaction between NadA and its receptor, decreased adhesion of NM on hBMECs, and reduced translocation of NM across BBB *in-vitro*. The selected NadA blocking VHHs could be promising molecules for therapeutic translation.

## Introduction

*Neisseria meningitidis* (NM) causing invasive meningococcal disease (IMD) is predominantly noticed in infants and young adults with higher rates of fatality, while survivors exhibit long term sequelae (ECDC European centre for disease prevention and control, 2015). There are 13 serogroups of NM differentiated based on biochemical composition of polysaccharide capsule, however, only A, B, C, W, and Y serogroups cause invasive outbreaks throughout the world (Harrison et al., 2009). Although the licensed prophylactic vaccines have reduced the prevalence of fatal IMD in the recent years (Pelton, 2016; Presa et al., 2019), meningitis caused by NM remains a medical emergency and therefore warrants standard therapy of antibiotics such as cephalosporin, vancomycin, and ceftriaxone (Hoffman and Weber, 2009). However, few reports have indicated decreased susceptibility of NM to antibiotics (Jorgensen et al., 2005; Gorla et al., 2018; Zouheir et al., 2019). Besides, antibiotics can promote the production of bacterial debris like lipopolysaccharide, peptidoglycan, and DNA during the therapy culminating TLR pathway activation in microglia leading to indirect neurotoxicity and tissue damage (Fischer and Tomasz, 1984; Lehnardt et al., 2003, 2008). Therefore, alternative strategies are being explored to treat such serious infectious diseases.

Monoclonal antibody based therapy has gained acceptance in treating cancer, chronic inflammatory disease, infectious and neurodegenerative diseases due to their specificity and potency (Cai, 2017). On the other hand, single-domain antibodies also known as nanobodies are better performers than conventional antibodies because of their small size (15kDa), stability in harsh condition (e.g., extreme pH and high temperatures) and possibility to produce in bacterial and yeast expression system (Hamers-Casterman et al., 1993; Muyldermans et al., 2009). Moreover, the antigen-binding loop of nanobody is dominated by protruding CDR3 that forms a convex paratope (de Genst et al., 2006). These features help nanobodies to reach even those receptor clefts or binding pockets that are inaccessible to conventional antibodies. Although the nanobodies are derivative of camelid heavy chain only antibodies, they are non-immunogenic to humans because of a high degree of identity with human variable heavy chain (Muyldermans, 2013).

In the present study, we have generated nanobodies (also known as VHH–variable heavy chain domain) against the trimeric auto-transporter adhesin of NM–*Neisseria* adhesin A (NadA). NadA is a potent immunogen, expressed in 50% of hypervirulent *Neisseria* (Comanducci et al., 2002) and forms one of the components of licensed vaccines capable of producing anti-neisserial antibodies in immunized mice (Pizza et al., 2000; Fagnocchi et al., 2013). NadA is a member of oligomeric coiled-coil adhesins (OCA), which structurally possess COOH–terminal membrane anchor (made of β-barrels), the N-terminal globular head like domain and the intermediate region of elongated coiled-coil stalk made of α-helix (Malito et al., 2014). Recently we have observed the binding ability of NadA to human brain microvascular endothelial cells (hBMECs) and the receptor-binding pockets were found in the globular head (residues A^33^ to K^69^) and coiled-coil domain (residues L^121^ to K^158^) of NadA protein (Kánová et al., 2018; Mertinková et al., 2020). To produce ligand-blocking specific nanobodies, synthetic analogs of the receptor-binding pockets of NadA were used for biopanning in the phage display as opposed to the entire protein.

Phage display is a powerful tool to isolate antibodies with high specificity from a diverse pool of variant antigens. Antibodies are expressed as fusion proteins to phage coat proteins. Among the various coat proteins, pVIII (the most abundant coat protein) and pIII (essential for phage infection) are employed to produce fusion targets (Brunet et al., 2002). pVIII of phage is used to display short peptides as it is sensitive to the length of foreign peptide insert (Iannolo et al., 1995). On the contrary, large proteins can be displayed on more tolerant pIII (Pavoni et al., 2013).

To block the receptor-binding pockets of NadA by VHH, the Hyperphage system (M13KO7ΔpIII) was used to superinfect *E. coli* transformed with phagemid-pJB12 carrying VHH sequences. The resulting VHH-phage library was subsequently panned with synthetic analogs of receptor-binding pockets of NadA viz., NadA-gd^A33−K69^ (spanning A^33^ to K^69^ of globular head) and NadA-cc^L121−K158^ (spanning L^121^ to K^158^ of a coiled-coil domain). Specific phages eluted after panning were overexpressed to produce soluble VHH. Next, functional assays were performed to evaluate the abrogation of interaction between NadA and hBMEC, and adhesion of NM on hBMECs. VHH produced against NadA-gd^A33−K69^ and NadA-cc^L121−K158^ in this study could be extended for its potential use against NM infection.

## Materials and Methods

### Production of Recombinant 6x His-NadA

**Figure 1:**
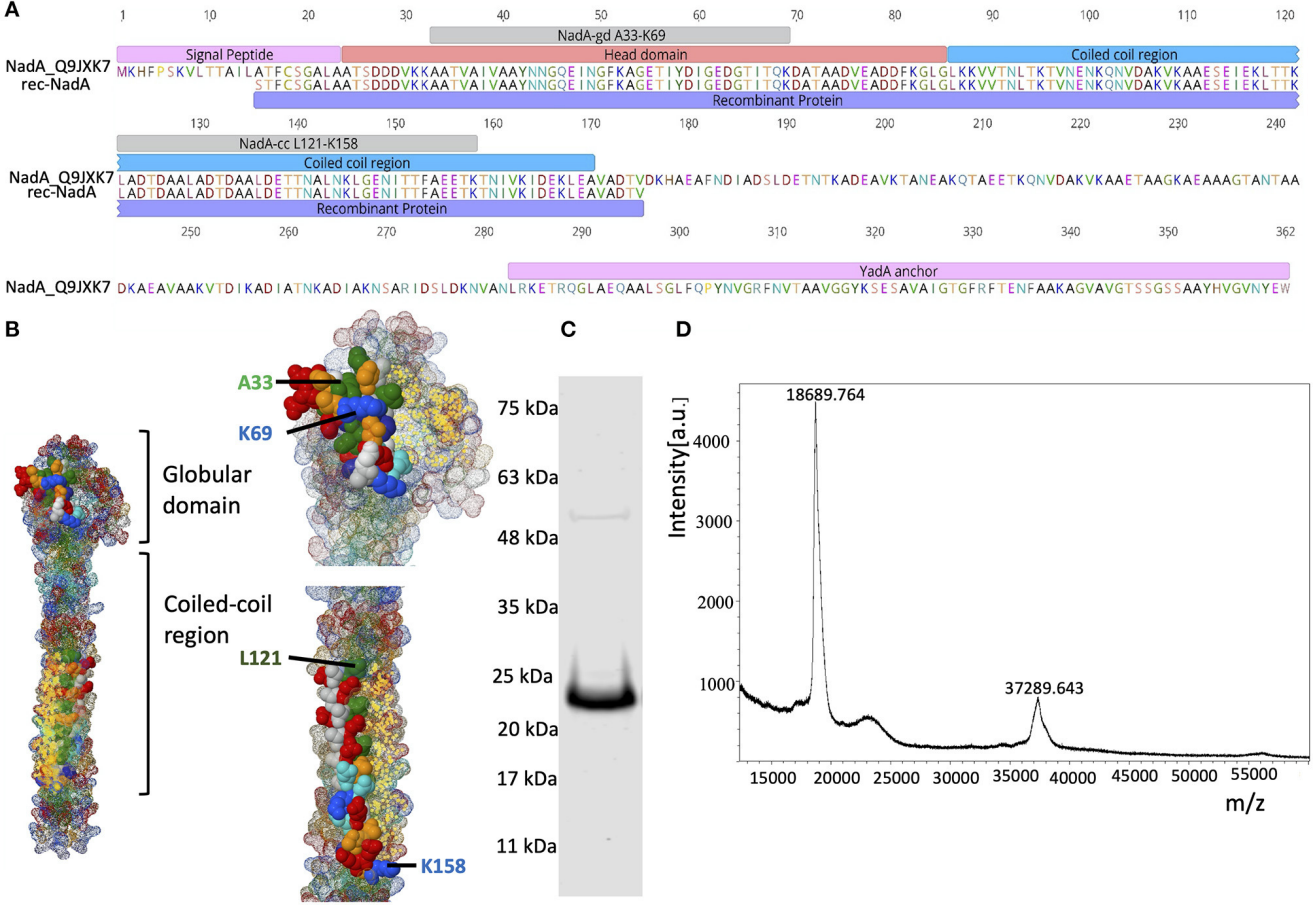
Domain organization of NadA and purity of rec-NadA. **(A)**–Schematics of NadA domains–signal peptide, head domain, coil-coiled region, transmembrane–YadA anchor are marked on the amino acid sequence alignment. An alignment is shown between Uniprot ID: Q9JXK7 and the sequence amplified from NM, which was used to construct rec-NadA. Receptor-binding sites on NadA (NadA-gd^A33−K69^ and NadA-cc^L121−K158^) are also depicted. **(B)**–Crystal structure of NadA protein (globular domain and coil-coiled region) is illustrated emphasizing hBMEC receptor-binding regions–NadA-gd^A33−K69^ and NadA-cc^L121−K158^. **(C)**–Purified rec-NadA resolved on SDS PAGE. **(D)**–Molecular mass of the rec-NadA analyzed by MALDI-TOF. It is noteworthy that a protein band of 24 kDa is observed in SDS PAGE, while in mass spectrometry the molecular mass was 18.68 kDa. The presence of NadA dimers is also noticeable in both SDS PAGE and MALDI-TOF. NadA_Q9JXK7–Uniprot accession number from NadA.

A gene fragment of NadA (GenBank–NP_274986) encoding globular head domain (A^24^ to G^85^) and first domain of coiled-coil region (L^86^ to A^170^, details of the sequence are in Figure 1) was amplified from genomic DNA isolated from NM (Strain MC58, isolate–M1/03). Details on primers (NadA F and NadA R primers) and amplicon length are presented in Table 1. PCR product was digested with BamHI and KpnI enzymes and ligated into in-house modified pQE-30-mCherry-stop-GFP plasmid as described earlier (Mertinková et al., 2020). The cloned vector was electroporated into *E. coli* M15 strain (Qiagen, Germany). Clonal selection on LB-carbenicillin agar plate (LB broth, Sigma Aldrich, Germany, Carbenicillin, 50 μg/mL, Duchefa Biochemie BV, Haarlem, The Netherlands), overexpression of the recombinant protein, its purification with nickel affinity chromatography (Ni-NTA agarose beads, ABT, Spain) followed by anion exchange [Bis-Tris, pH 6.0 containing 8M urea (Sigma Aldrich) for binding; Bis-Tris, pH 6.0 with NaCl (Sigma Aldrich) gradient for elution] was performed as described in our earlier publications (Jiménez-Munguía et al., 2018; Kánová et al., 2019). The purified protein was stored in 10 % glycerol (MikroChem spol. SRO, Pezinok, Slovakia) at −20°C in several aliquots.

**Table 1:** Primers used in the study.

**No**.	**Description**	**Primer name**	**Sequence (5^**′**^–3^**′**^)**	**Amplicon length (bp)**
1	Rec-NadA production-	NadA F	AAAGGATCCACTTTCTGTAGCGGCGCACTG	498
		NadA R	AAAGGTACCGACGGTATCAGCCACGGCTTC	
2	Reverse transcription	sdAb-Not-R	CCAGCGGCCGCTSWGGAGACRGTGACCWGGGTCC	–
3	Amplification of VHH	NB-F	GCGGCCCAGCCGGCCGCCSAGGTGSAGGTSSWGSMGTC	~500
		NB-R	AAAGGCCCCCGAGGCCGATSWGGAGACRGTGACCWGGGTCC	
4	Insert check	UA Insertom F	CGCATCACCATCACCATCACG	~540
		UA Insertom R	ACCAAAATTGGGACAACACCAGTG	
5.	qPCR	NadAqPCR-F	CGACAGCTTGGACAAAAACGT	122
		NadAqPCR-R	CAGCCGTTACATTGAACCGAC	
6	Amplification of region upstream of NadA promoter	Nad-N2	TAAGACACGACACCGGCAGAATTG	~253
		Nad-SP	GCTCATTACCTTTGTGAGTGG	

*Underlined nucleotides denote restriction sites GGCCCNNNNGGCC–SfiI, GGATCC–BamHI, and GGTACC–KpnI*.

### Animal Welfare

Four years old healthy male llama *(Llama alpaca*) was purchased from local zoo (Kosice zoo) and reared at the animal house of the University of Veterinary Medicine and Pharmacy in Kosice. Anesthetizing llama, *in-vivo* immunizations, and blood collection were performed according to the guidelines of EU animal welfare legislation and the University ethical committee.

### *In-vivo* Immunization, RNA Isolation, and Reverse Transcription

*In-vivo* immunization of llama was performed following the protocol described by Pardon and co-workers with some modifications (Pardon et al., 2014). Briefly, 200 μg of rec-NadA was used for the first immunization followed by 5–weekly immunizations with 100 μg of the same protein (total 700 μg of protein used for immunization). For the first immunization, rec-NadA was mixed with Freund's complete adjuvant (1:1) (Sigma-Aldrich) and 1 mL of the mix was injected i/m in quadriceps femoris. Subsequent immunizations were performed using the emulsion of rec-NadA and Freund's incomplete adjuvant (1:1) (Statens serum institut, Copenhagen, Denmark). One week after the last immunization, 100 mL of blood was collected from the jugular vein in the presence of heparin (2500 IU) (Zentiva a.s., Czech Republic). Peripheral blood mononuclear cells (PBMC) were isolated from the heparinized blood using density gradient centrifugation in Histopaque medium (Sigma-Aldrich) as described in the manufacturer's instruction. RNA was isolated from the PBMC using the RNeasy mini kit (Qiagen) and reverse-transcribed with RevertAid (Thermo Scientific, Bratislava, Slovakia) following the manufacturer's instructions. sdAb-Not-R primer (Table 1) was used in the reverse transcription reaction.

### Amplification of VHH, Cloning of VHH in Phagemid, and Preparation of VHH-*E. coli* Library

VHH region was amplified by using degenerated primers NB-F and NB-R (Table 1), which flank the frameworks 1 to 4. As we aimed to obtain diversified VHH clones instead of amplifying single species of VHH sequence, PCR was performed on 500 ng of cDNA/reaction with 25 cycles (Thermal cycler XT^96^, VWR international, Bratislava, Slovakia). Cycling conditions (95°C–2 min and 25 cycles of 95°C–20 s, 56°C–30 s and 68°C–1 min) were sufficient to obtain merely visible amplicons on agarose gel electrophoresis. PCR cycles were set to 25 based on a pilot assay in which, 2.5 μL of the amplicon was tested on gel electrophoresis after 20, 25, 30, and 35 cycles.

Amplicons were gel purified (Macherey-Nagel, Germany) and digested with restriction enzyme SfiI (Thermo Scientific Bratislava, Slovakia) at 50°C for 1 h, ligated into SfiI digested phagemid pJB12 (kindly provided by Andreas Plückthun, Universität Zürich, Zürich, Switzerland) and electroporated into *E. coli* XL-1 blue (New England Biolabs, Germany) using a preset method for *E. coli –* in 1 mm cuvette (Cell Projects Ltd, UK), voltage: 1.8 KV, capacitance: 25 μf and resistance 200 Ω in Gene Pulser X cell^TM^ (Biorad, Watford WD, UK). Total 19 electroporations were performed and the transformed *E. coli* were grown in SOC medium (New England Biolabs, UK) for 1 h followed by plating them on 6 LB agar (Sigma Aldrich) plates (diameter 18.5 cm) supplemented with tetracycline (50 μg/mL (Duchefa Biochemie BV) and chloramphenicol (50 μg/mL (Duchefa Biochemie BV). Plates were incubated overnight at 37°C. Fifteen randomly selected transformants were sequenced using vector-specific primers UA-Insertom-F and R (Table 1) to confirm the presence of VHH sequence as well as to assess diversity among cloned VHH. Lastly, transformed *E. coli* colonies (VHH-*E. coli* library) were scraped and stored as aliquots of 10 mL of LB medium (Sigma Aldrich): glycerol (MikroChem) (1:1 V/V) at −80°C until further use.

### Phage Packaging

Two aliquots of frozen VHH-*E. coli* library (20 mL) were inoculated in 800 mL of 2xTY medium (triptose 16 g/L, yeast extract 10 g/L and NaCl 5 g/L, pH 7.0) supplemented with tetracycline 50 μg/mL (Duchefa Biochemie BV), chloramphenicol 50 μg/mL (Duchefa Biochemie BV) and 4% glucose (Duchefa Biochemie BV) to obtain the initial OD_600_ ~0.1. The culture was incubated for 8 h to reach the final OD_600_ = 0.5 which followed the addition of Hyperphage M13 K07ΔpIII (Progen Biotechnik, Germany) to superinfect VHH-*E. coli* library (MOI = 20 phages/*E. coli* cell). Next, the superinfected culture was incubated for 30 min at 37°C (no shaking) and centrifuged at 3,500 × *g* for 20 min (Hettich Germany; Model MIKRO 220) to eliminate unbound phages in the supernatant. The resulting pellet was resuspended in 2xTY medium (Sigma Aldrich) supplemented with tetracycline: 50 μg/mL (Duchefa Biochemie BV), chloramphenicol: 50 μg/mL (Duchefa Biochemie BV) and kanamycin: 50 μg/mL (Duchefa Biochemie BV) and incubated overnight with shaking (200 RPM) at 25°C. Escaped phages were precipitated in 20% polyethylene glycol (Sigma Aldrich, Germany), 2.5 M NaCl (Sigma Aldrich), and resuspended in phage dilution buffer (10 mM Tris-HCl pH 7.5, 20 mM NaCl, and 2 mM EDTA, Sigma aldrich). The enumeration of phages was performed by a spectrophotometric method by measuring the absorbance at A_269_ and A_320_. The number of phages per mL was calculated using the formula [(A_269_–A_320_^*^ 6x10^16^)/(no of bases per virion)] (Phage Concentration Calculator, 2012).

### Negative Adsorption

Purified phages (1 × 10^14^) were first incubated for 1 h in empty tubes to eliminate plastic binders. Unbound phages were then incubated for 1 h in 1.5 mL centrifuge tubes coated with 2% bovine serum albumin (BSA) (Merck, Germany), and transferred to another tube containing SpeedBeads^Tm^ magnetic neutravidin coated particles (GE health care, USA) for 2 h. After incubation, the tube was placed on the magnetic separator and the flow-through containing phages (this is the phage library carrying VHH, hereafter designated as VHH-phage library) was collected for biopanning.

### Infectivity Assay of Escaped Phages

To confirm the inability of escaped phages to infect *E. coli*, 10 μl of VHH-phage library was used to challenge 50 mL of *E. coli* XL-1 blue (OD_600_ 0.45) for 30 min at 37°C. The challenged *E. coli* XL-1 blue were then plated on the LB (Sigma Aldrich) agar plate supplemented with Tetracycline: 50 μg/mL (Duchefa Biochemie BV), chloramphenicol: 50 μg/mL (Duchefa Biochemie BV) and Kanamycin: 50 μg/mL (Duchefa Biochemie BV).

### Biopanning

Receptor binding pockets of rec-NadA–NadA-gd^A33−K69^ and NadA-cc^L121−K158^ was recently identified in our previous publication (Mertinková et al., 2020). The synthetic analogs of peptides–NadA-gd^A33−K69^ and NadA-cc^L121−K158^ containing biotin tag at C-terminus (Supplementary Table 1) were dissolved in 5 m Urea (AppliChem, Germany), captured separately on neutravidin coated magnetic beads and incubated with VHH-phage library (1 × 10^14^ Phages) diluted in 1X PBS (1.5 mL) for 1 h at 4°C with constant shaking. Next, the tubes were placed on the magnetic separator, flow-through was discarded and beads were washed with PBS-Tween 20 (0.1%, Sigma Aldrich) (PBS-T). The second washing was performed at 4°C for 16 h. Nine more washings were performed with PBS-T for 5 min and with a change of tubes at every wash. Finally, phages were eluted in 100 mM glycine–HCl (pH 2.7, MikroChem spol. SRO, Bratislava), and pH was raised to 7.5 immediately with Tris (5M, AppliChem). The single round of biopanning was deliberately performed to preserve the diversity of phages and increase the chances of retaining blocking nanobodies.

### Amplification of the VHH Gene From Eluted Phages, Its Ligation, and Overexpression of the Protein

Eluted Phages were used to amplify VHH using NB-F and NB-R primers (Table 1) with cycling conditions of PCR: 95°C-2 min, 30 cycles of (95°C-20 s, 56°C-30 s, 68°C-1 min), 68°C-10 min. The amplified VHH was digested with SfiI at 50°C for 1 h, column purified (Macherey-Nagel, Germany), ligated into in-house modified vector–pQE30-UA mCherry-Stop-GFP (Supplementary Figures 1A,B) and electroporated into *E. coli* Shuffle (New England Biolabs, UK) using aforementioned electroporation conditions. Three electroporations were performed per elution and the transformed *E. coli* were primarily grown on SOC medium for 1 h (300 RPM) followed by the spread plating on 3 LB agar plates supplemented with carbenicillin (100 μg/mL) (Duchefa Biochemie) at 30°C overnight.

Forty-eight clones carrying VHH against each peptide (NadA-gd^A33−K69^ or NadA-cc^L121−K158^) were picked randomly and resuspended in 1 mL of TB medium [tryptone: 12 g/L (Duchefa Biochemie), yeast extract: 24 g/L (Duchefa Biochemie), glycerol: 0.6% (MikroChem), 100 mL/L of Na_2_HPO_4_. 12H_2_O (25 mM, Sigma Aldrich), KH_2_PO_4_ (25 mM, Sigma Aldrich) and 20 mL/L glucose (Duchefa Biochemie)] supplemented with carbenicillin (100 μg/mL, Duchefa Biochemie). Transformants were propagated for 16 h at 30°C with constant shaking (250 RPM). One hundred μL of each culture were conserved as glycerol stock, 10 μL of each culture was used for DNA extraction (98°C for 10 min) and the remaining culture was centrifuged at 4,000 RMP for 40 min. The supernatant was discarded and the bacterial pellet was dissolved in 1 mL TB medium devoid of glucose but supplemented with 0.5 mM IPTG (Thermo Scientific). Induction was carried out at 30°C for 3 h (250 RPM) followed by 16 h at 22°C (shaking 250 RPM). The induction of VHH was judged by monitoring non-fused GFP epifluorescence. After induction, bacteria were centrifuged at 4,000 RPM for 40 min, the pellet was resuspended in lysis buffer (50 mM NaH_2_PO_4_.2H_2_O, 300 mM NaCl, 8M Urea, 10% glycerol, 10 mM Imidazole, pH 8 subjected for 4 freeze-thaw cycles and 10 rounds of sonication (45-s burst at 75 Hz, 1 min pause). The lysate was centrifuged at 12,000 RPM for 30 min and the supernatant was recovered.

### Binding of VHH to Peptides–NadA-gd^A33-K69^ and NadA-cc^L121-K158^ (Dot Blotting)

One mL of bacterial lysate from all the 96 clones (48 clones carrying VHH against NadA-gd^A33−K69^ and 48 clones carrying VHH agaisnt NadA-cc^L121−K158^) were purified by nickel affinity chromatography using His-Mag sepharose Ni beads (GE Healthcare) according to manufacturer's instructions. One μL of purified VHH was spotted on the nitrocellulose membrane (GE healthcare). Lysate of the empty *E. coli* SHuffle was also spotted on the membrane for negative control. The membrane was blocked with 5% BSA in Tris-buffered saline containing Tween 20 (0.05%, TTBS) (Sigma) for 1 h and further incubated for another hour with synthetic analogs of–NadA-gd^A33−K69^ / NadA-cc^L121−K158^. After three washings (5 min each) with TTBS, streptavidin HRP conjugate (1: 30,000) (Thermo Scientific) was added on the membrane (1 h incubation) followed by repeated washing with TTBS (five times). Lastly, SuperSignal West Dura substrate (Thermo Scientific) was added and the chromogenic reaction was documented on C-digit (Li-cor, USA).

### Blocking the Interaction Between rec-NadA and Receptor of hBMECs

Culture of the hBMECs (D3 cell line, Merck/Millipore), and extraction of the membrane proteins was performed as described in our previous publication (Jiménez-Munguía et al., 2018). Four hundred micrograms of hBMEC proteins were separated on SDS-PAGE and transblotted on a nitrocellulose membrane (Supplementary Figure 2A). The membrane was cut vertically to obtain strips of 0.3 cm and they were subjected to western blot with 5 μg of rec-NadA as described previously (Mertinková et al., 2020). The specific interaction of rec-NadA with ~15 kDa receptor of hBMECs was reported (Supplementary Figure 2B). In the present study, VHH targeting NadA-gd^A33−K69^ and NadA-cc^L121−K157^ were tested either individually or in combination to block the interaction of rec-NadA with ~15 kDa hBMEC proteins (possible receptor). Briefly, 125 ng (7 pM) of rec-NadA was mixed with 600 ng (42 pM) of purified VHH targeting either NadA-gd^A33−K69^ / NadA-cc^L121−K158^ or the combination of both VHHs in 200 μL of 1x TBS (pH 7.2). VHH–rec-NadA was mixed for 1.5 h with constant shaking at room temperature. The pre-blocked rec-NadA was incubated for 1.5 h on membrane strip retaining the portion of ~15 kDa hBMEC protein (transblotted). After three-time washings with TTBS, strips were incubated with anti-His antibody (1:5000) (Thermo Scientific) for 30 min at room temperature. After two washings with TTBS, SuperSignal West Dura was added and signals were captured on C-digit. Strips incubated with rec-NadA (without pre-blocking) or just VHH (without rec-NadA) served as positive and negative controls, respectively.

### Blocking the Adhesion of rec-NadA on hBMEC Cells–Immunocytochemistry (ICC)

Low passage D3 cells were grown on type IV collagen (Sigma-Aldrich, St. Louis, MO) coated coverslips in 12 well cell culture plate in EBM2 medium (Lonza, Basel, Switzerland) up to 70 % confluency as described previously (Jiménez-Munguía et al., 2018). Cells were washed with fresh EBM2 medium (without FBS) and incubated with either 20 μg of rec-NadA (positive control) or 20 μg of rec-NadA pre-blocked for 2 h at room temperature with various concentrations of VHH- VHH_B5_ and VHH_G9_ (20, 4, and 60 μg each) or VHH_F3_ and VHH_G9_ (20, 40, and 60 μg each). Subsequently, 4 washings with PBS (pH 7.2) were performed prior to and after fixing the cells with 4% paraformaldehyde (Merck) for 15 min at room temperature. Thereafter, the cells were incubated with anti-6x His rabbit polyclonal antibody-FITC conjugate (1:500 in PBS with 1% BSA) (Abcam) for 1 h and three washes with PBS were performed. Lastly, mounting the coverslips on a glass slide was performed with Fluoroshield^Tm^ containing DAPI (Sigma Aldrich). For negative control, cells were either incubated with just the VHH_B5+F3+G9_ (60 μg) or the anti-6x His antibody-FITC. Additional control was included in the assay in which cells were incubated with 20 μg of rec-NadA pre-treated with 20 μg of VHH recognizing envelope protein of West Nile virus (non-related VHH).

### Large-Scale Production of VHH_B5_, VHH_F3_, and VHH_G9_

Transformants from glycerol stock producing VHH_B5_, VHH_F3_, and VHH_G9_ were propagated and induced in 100 mL of TB medium as described above. Bacterial lysate containing VHH was purified by nickel affinity chromatography using Ni-NTA agarose beads (Jena Bioscience, Thuringia, Germany) followed by cation exchange chromatography and gel filtration on Äkta purifier (GE Health care) as described before (Kánová et al., 2019).

### Blocking the Adhesion of NM to hBMECs: Quantification by qPCR

Overnight grown NM (cultured in brain heart infusion broth supplemented with 10 mM MgCl_2_, Sigma Aldrich, 60 mL,) was centrifuged (6000 RMP for 10 min) and the pellet was washed with DMEM with high glucose medium (Biowest, Nuaillé–France). In overnight culture, NM are supposed to be in a stationary growth phase (doubling time 40 min, Tobiason and Seifert, 2010), which should support high expression of NadA (Comanducci et al., 2002; Metruccio et al., 2009). Additionally, DNA extracted from the overnight cultured NM was used to amplify the tetranucleotide tract (TAAA) upstream of the *nadA* promoter using previously published Nad-N2 and Nad-SP primers (Table 1) (Metruccio et al., 2009). This tetranucleotide tract was shown to control the phase variable expression of NadA (Martin et al., 2003; Metruccio et al., 2009). The amplified region–upstream of NadA promoter (~253 bp) was sequenced using Nad-N2 primer that has identified 13 TAAA repeats (Supplementary Figure 3) leading to high levels of NadA expression (Martin et al., 2003; Metruccio et al., 2009). NM were killed in 4% formaldehyde for 15 min, washed and stained with acridine orange (5 mg/mL, Sigma aldrich) for 5 min. Bacteria were washed and enumerated on single laser flow cytometer (BD Accuri^Tm^ C6) using the following parameters–flow rate: slow with 20 μL/min, a threshold to avoid noise: 80,000 on forward scatter (FSC-H), channel 1, side scatter (SSC-A).

One million NM (harvested from overnight culture) either incubated for 1 h with the combination of VHH_F3_ and VHH_G9_ (each 160pM resuspended in 150 mL of 1X PBS, pH 7.2; blocked NM) or PBS (positive control) were exposed to ~ 2.5 × 10^5^ hBMECs (MOI = 1:4) in 6 well-plate for 1 h at 37°C. Alternatively, hBMECs incubated for 1 h at 37°C without any exposure of NM served as the negative control. It is noteworthy that no antibiotics were used during the incubation of the cell with NM. After the incubation, cells were washed three times with DMEM medium and subjected for DNA extraction. Assay was performed in duplicate.

Simultaneously, the dilution series of NM was generated to plot the standard curve for enumeration of bacterial count based on DNA copy number vs log_10_ of the number of NM spiked in the cells. In short, 2 × 10^6^ NM (harvested from overnight culture and counted on flow cytometer) were 4-fold diluted up to 30 bacteria in 150 mL of PBS (total dilutions = 8, Supplementary Table 2). NM from dilution series were then exposed to ~ 2.5 × 10^5^ hBMECs in 6 well-plates Immediately after spiking, DNA was extracted (using DNeasy kit, Qiagen) from the cells. Primers targeting NadA gene–NadAqPCR-F and NadAqPCR-R (Table 1) were used to quantify DNA copy number in 50 ng of extracted DNA. Note that the entire assay was performed in duplicates.

Quantitative real-time PCR (qPCR) was performed on StepOnePlus (Thermo Fisher Scientific, USA) as described in our earlier publication (Kánová et al., 2019). In short, 50 ng of DNA (2 μL), was used in 1x qPCR GreenMaster with highROX (Jena Bioscience, Germany) master mix (16 μL) contain 10 pMol NadA primers (1 μL of forward and reverse each). Amplification conditions were–95°C–10 min, 30 cycles of (95°C–15 sec, 60°C–30 s, 72°C for 30 s). A melt curve was generated at the end of the PCR reaction from 60°C to 95°C with 0.3% temperature increment/sec. For quantification, a standard curve was plotted between the log_10_ of NM (X-axis) and Ct values (Y-axis). Using the formula X = (y-c)/m where c is the y-intercept on X-axis and m is the slope. The number of bacteria in hBMECs exposed to either blocked or unblocked NM was determined using this plot. One-tailed Mann-Whitney *U*-test at 95% confidence limit was employed using Graphpad Prism v 5 (GraphPad Software Inc, CA, USA) to determine the statistical significance between the NM adhered on hBMECs in three technical replicates.

### Blocking the Traversal of NM Through *in-vitro* BBB Model

hBMECs were grown in 0.4 μM Transwell® inserts (Becton Dickinson, New Jersey, USA) as described earlier (Majerova et al., 2019). In brief, the inserts were coated with collagen type IV (10 μg/cm^2^ (Sigma-Aldrich) and 5 μg/cm^2^ fibronectin (Sigma- Aldrich) before seeding of hBMECs (12 000 cells/filter) in the luminal chamber. The cells were allowed to grow in EBM-2 medium (Lonza, Basel, Switzerland) containing 15% fetal calf serum (Thermo Scientific), 2 mM L-glutamine (Life Technologies, Carlsbad, CA), 10 μg/mL ascorbic acid (Sigma-Aldrich), 550 nM hydrocortisone (Sigma-Aldrich), 2.5 μg/mL insulin (Sigma-Aldrich), 2.5 μg/mL transferrin (Sigma-Aldrich), 2.5 ng/mL sodium selenite (Sigma-Aldrich), 50 μg/mL gentamicin (Sigma-Aldrich), BME vitamins, and BulletKit SingleQuots (Lonza, Basel, Switzerland). After seven days, TEER value between luminal and abluminal chambers was measured as 300 ± 20 Ω cm^2^ with Ohm meter “EVOM” (World Precision Instrument, EVOM Sarasota, FL, USA), to confirm the integrity of the BBB.

NM culture (overnight grown culture) was centrifuged, the pellet was washed with DMEM with high glucose medium (Biowest, France) and stained with acridine orange (1 μg in 1 mL PBS, pH 7.2) (Sigma Aldrich) for 5 min. Bacteria were enumerated with the flow cytometer. One million stained NM were preincubated with the combination of VHH_F3_ and VHH_G9_ (each 166.5 pM) for 1.5 h at room temperature. Preincubated NM were added to the luminal chamber of the insert (Figure 5). After incubation (1 h at 37°C), 500 μl of the medium from both luminal and ab-luminal chambers were enumerated with flow cytometry as described above. Stained NM without VHH preincubation served as the positive control. BBB model without NM incubation served as the negative control. The assay was performed in triplicates. Statistical significance was determined using unpaired *t*-test with Welch correction using Graphpad Prism v 5.

## Results

### VHH Amplification, Phage Packaging, and Escaping

The recombinant form of NadA (rec-NadA), spanning 161 amino acids from S^1^ to V^175^ was produced in *E. coli* M 15 strain (Qiagen, Germany) that encompasses both globular head domain (A^24^ to G^85^) and the first coiled-coil domain (L^86^ to A^170^) (Figures 1A,B). On the SDS-PAGE rec-NadA appeared at 24 kDa (Figure 1C), whereas, using MALDI-TOF its molecular mass was confirmed as 18.6 kDa with 37.2 kDa dimer (Figure 1D). The molecular mass of monomer observed with MALDI-TOF matched with the predicted mass of rec-NadA in Genious pro software (Data not shown). Purified rec-NadA was then used for immunization.

The VHH region (~500 bp) was amplified from cDNA of immunized llama (RNA isolated from B cells) using 25 cycles of PCR to retain the maximum possible diversity of the VHH gene. The amplified VHH was digested and ligated into pJB12 phagemid. It should be noted that the pJB12 phagemid used for ligation was predigested with SfiI and agarose gel electrophoresis (0.7% gel, 120 V) was performed for 5 h to eliminate carryover of undigested phagemid (Supplementary Figures 4A,B). In total 19 electroporations were performed, 100 ng of ligation mix was used for each electroporation. Transformants growing on 6 LB plates (lysogeny broth plates, 58 cm^2^) supplemented with tetracycline (50 μg/mL) and chloramphenicol (50 μg/mL) (Supplementary Figure 4C) were scrapped in LB medium containing 50% glycerol (VHH–*E. coli* library) and used for phage packaging. *E. coli* electroporated without DNA or with digested phagemid (negative controls) had no colonies after 16 h of incubation on antibiotic-containing LB plates.

**Figure 2:**
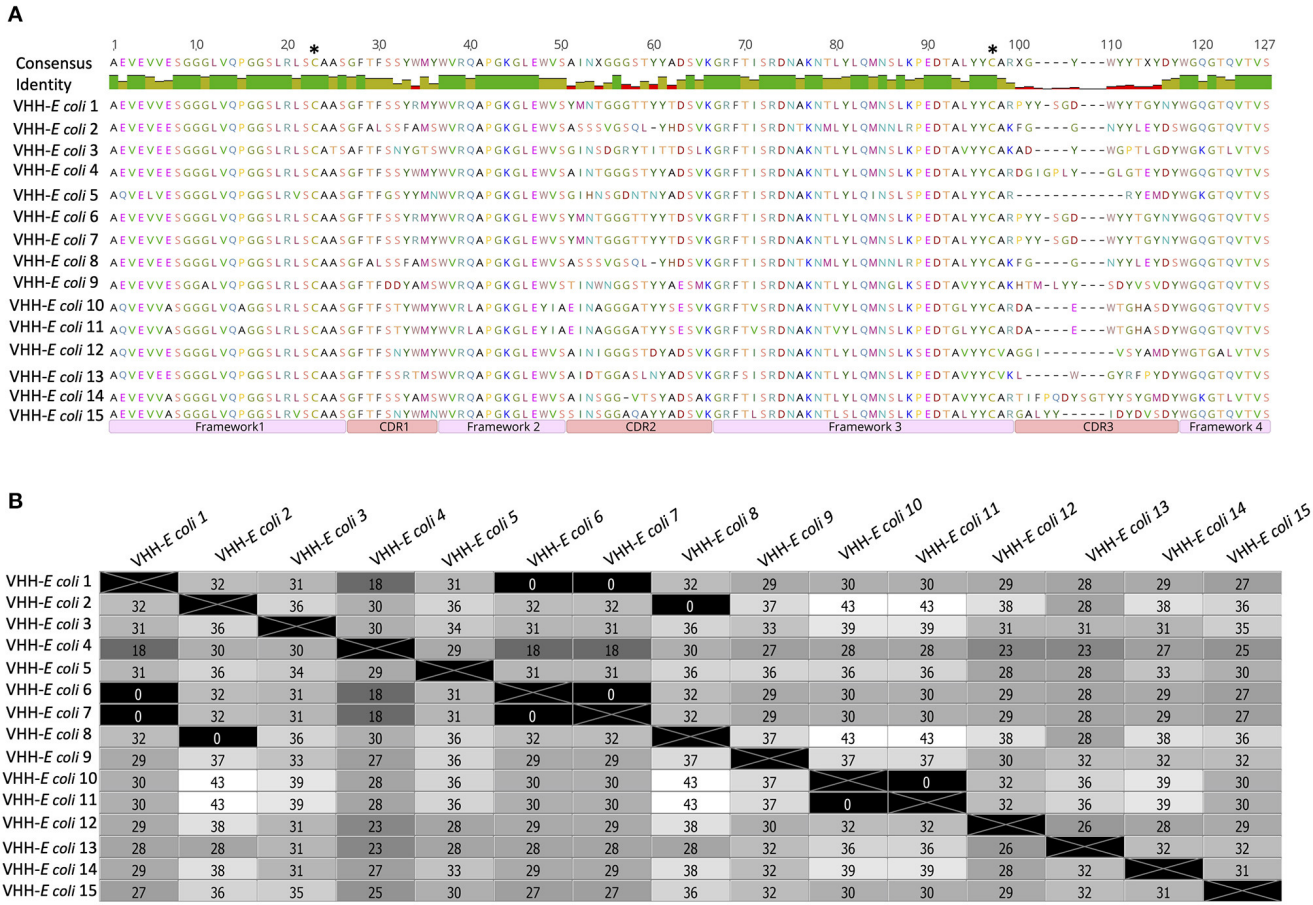
Amino acid sequences of VHH amplified from the transformants of VHH-*E. coli* library. **(A)**–Sequences of 15 randomly selected clones were aligned and regions of framework and CDRs are marked. Two cysteine residues (C21 and C97) that can mediate the formation of a disulfide bond are marked with asterisks. The similarity of residues within the framework region 1–3 and high variation of residues in CDR regions: 1–3 is noticeable in the consensus. **(B)**–A distance matrix plot generated on the sequences from **(A)**. Number of amino acid residues differing in clone comparison is mentioned in each box. Various shades of gray correspond to the difference in residues in which white represents maximum difference and black represents no difference.

VHH from fifteen randomly picked clones from the VHH–*E. coli* library were sequenced and their amino-acid sequences were aligned to define CDR and framework regions (Figure 2A). A distance matrix was plotted based on amino-acid sequences (Figure 2B). The matrix showed that 3 clones (clone numbers 2, 6, and 7) carry similar VHH sequence. Likewise, clone numbers 10 and 11 had similar VHH sequence. All other clones were having a unique VHH sequence (Figure 2). VHH–*E. coli* library was inoculated in 800 mL of 2xTY medium to obtain initial OD 0.2 and the culture was incubated with constant shaking until the final OD = 0.45. *E. coli* culture was then superinfected with Hyperphage M13 K07ΔpIII for packaging of the phage particle carrying PIII fused with VHH (VHH–phage library).

### Packaged Phages Were Non-infectious to *E. coli*

The packaging strategy used here was to produce non-infectious phages once they escape from the *E. coli*. To achieve this, a super-short version of PIII present in pJB12 phagemid (containing only C terminal domain of PIII, CT) was used in combination with hyperphage M13 K07ΔpIII, which is devoid of PIII. Resulting packaged phage offspring should contain only the CT domain carrying VHH (Supplementary Figure 5).

As shown in Supplementary Figure 5 wild type PIII consists of N1, N2, and CT domains with flexible glycine-rich linkers (G1 and G2). The N2 domain is necessary to bind F pilus of *E. coli*, whereas the N1 domain is required to form a complex with the C-terminal domain of tolA at later stages of the infection process. The C-terminal domain is required for the release of viral particles from the host bacterial membrane. Thus, in the present study lack of N1 and N2 domains in the packaged phages should confer the inability to infect *E. coli*.

*E. coli* XL-1 blue when infected with packaged phages from VHH-phage library and spread on LB agar plate (supplemented with tetracycline, chloramphenicol, and kanamycin) showed no colonies after 16 h confirming non-infectivity of packaged offspring.

### Selection of VHHs Having Affinity to the Receptor-Binding Sites of NadA

VHHs that have an affinity to the receptor-binding site on NadA were selected with panning. Synthetic analogs of the receptor binding sites–NadA-gd^A33−K69^ and NadA-cc^L121−K158^ (Figures 1A,B, and Supplementary Table 1) were used for panning. SpeedBeads^Tm^ coated with either NadA-gd^A33−K69^ or NadA-cc^L121−K158^ were incubated with a VHH-phage library, unbound phages were washed rigorously and bound phages were eluted. Since the packaged phages produced in this study are unable to infect *E. coli*, PCR was used to amplify the VHH from eluted phages (Supplementary Figure 1A). Amplicons of ~ 500 bp were cloned into pQE30-UA mCherry-stop-GFP vector (Supplementary Figure 1B) and electroporated into *E. coli* SHuffle strain. A total of 48 transformants expressing VHH against each peptide (NadA-gd^A33−K69^ and NadA-cc^L121−K158^) were picked separately. The presence of GFP (non-fused) enabled us to monitor and standardize induction conditions (data on various trials of induction is not provided). The optimum protein production was achieved at 30°C for 3 h followed by 16 h incubation at 22°C, 250 RPM shaking, and 0.5 mM IPTG/mL concentration.

### Binding of VHH to NadA Peptides

**Figure 3:**
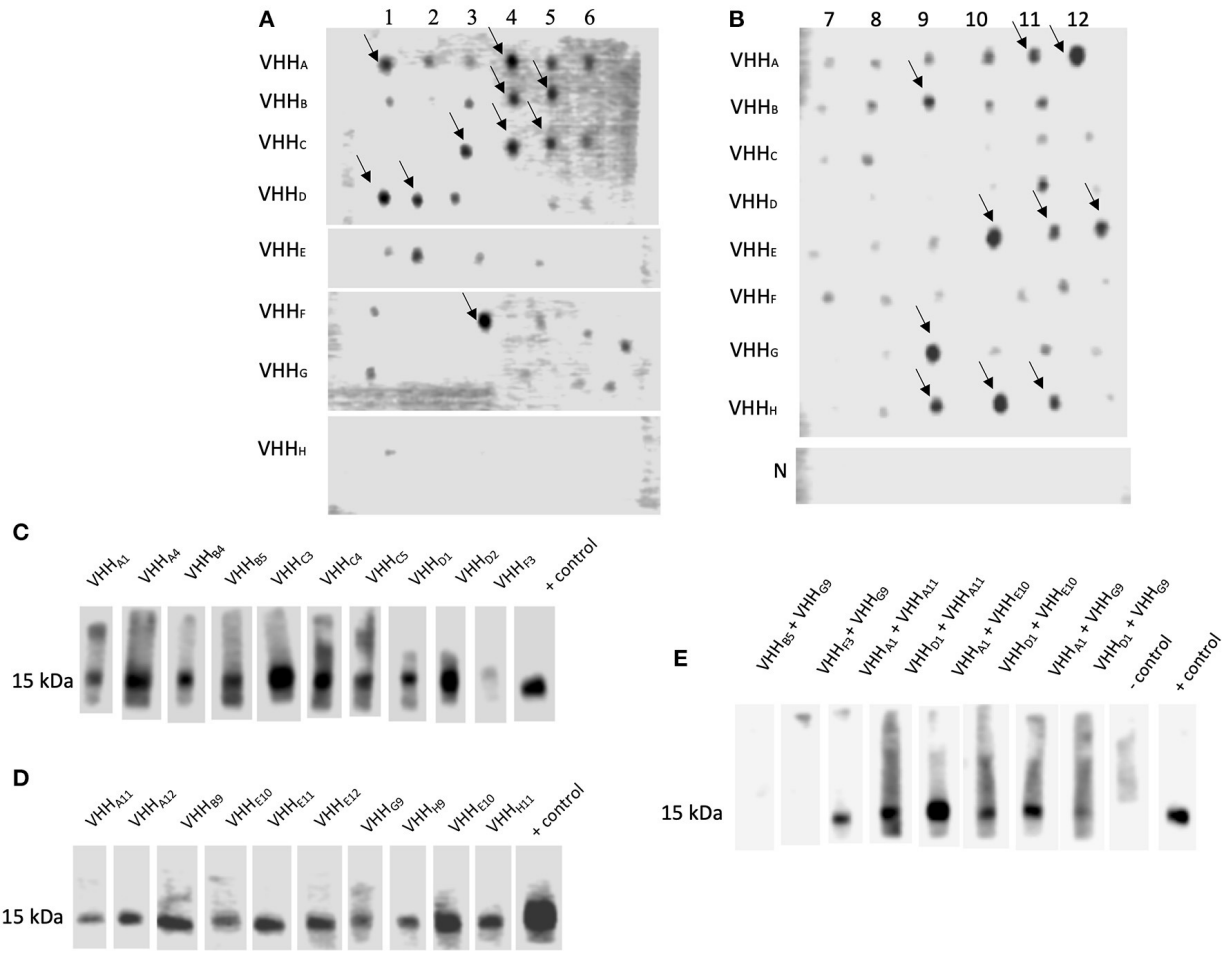
VHH Interacting with NadA peptides could block the interaction between rec-NadA and proteins of hBMEC. **(A,B)**–Ni-NTA purified lysate from the transformants spotted on the NC membrane was incubated with either NadA-gd^A33−K69^ **(A)** or NadA-cc^L121−K158^ **(B)** and their interaction was detected by streptavidin-HRP. Strong interactions are marked by arrows. Lysate of empty *E. coli* SHuffle (negative control) was subjected to similar dot-blot assay–row N in **(B)**. **(C–E)**–NC strips having 15 kDa receptor of hBMEC (transblotted) was incubated with pre-blocked rec-NadA. Preincubated was performed to block receptor binding site on rec-NadA by VHH selected against either NadA-gd^A33−K69^ **(C)** or NadA-cc^L121−K158^ **(D)** or combination of VHH selected against NadA-gd^A33−K69^ and NadA-cc^L121−K158^ **(E)**. The interaction was detected by the anti-His antibody. Likewise, strip incubated with rec-NadA without preincubation served as the positive control. The omission of rec-NadA served as the negative control.

Dot blot assay was performed on all clones to select NadA-gd^A33−K69^and NadA-cc^L121−K158^ binding VHH. Although several clones reacted with NadA peptides (Figures 3A,B), 20 VHH clones showing strong interaction with NadA peptides were chosen for further analysis. The specificity of the assay was confirmed by the absence of any dot-blot reaction for the lysate of empty *E. coli* SHuffle (Figure 3B).

### Blocking the Interaction Between rec-NadA and the Protein of hBMECs

In our recent study it was shown that rec-NadA interacts with hBMEC, mediated through ~15 kDa receptor [(Mertinková et al., 2020), Supplementary Figures 2A,B]. One of the aims of the present study was to block this interaction using VHH. Before the experimental blocking of the adhesion of NadA to hBMECs, the minimum concentration of rec-NadA required to detect visible interaction to the endothelial receptor was assessed. In the western blot targeting ~15 kDa receptor, it was observed that a minimum of 125 ng of rec-NadA was necessary to detect the interaction (Supplementary Figure 2C).

To block the adhesion of rec-NadA to the receptor of hBMECs, rec-NadA was preincubated with each VHH (total 20 different VHH were assessed), and then allowed to interact with ~15 kDa protein of BMECs in the Western blot analysis. Binding of the rec-NadA to ~15 kDa receptor was not abolished completely when rec-NadA was preincubated with VHH raised against either NadA-gd^A33−K69^or NadA-cc^L121−K158^ (Figures 3C,D). This might be because of two distinct receptor binding pockets (one on the globular domain and another on first coiled-coil) present on the NadA, which might interact independently with the receptor. Thus, blocking either of the sites was not sufficient to abrogate the interaction between NadA and the receptor. Therefore, both the receptor binding pockets of NadA were blocked with a combination of VHH raised against NadA-gd^A33−K69^ and NadA-cc^L121−K158^. Among all combinations, VHH_**B5**_ + VHH_**G9**_ and VHH_**F3**_ + VHH_**G9**_ could completely inhibit the binding of rec-NadA to the ~ 15 kDa receptor (Figure 3E).

### Blocking the Interaction of rec-NadA and hBMEC

**Figure 4:**
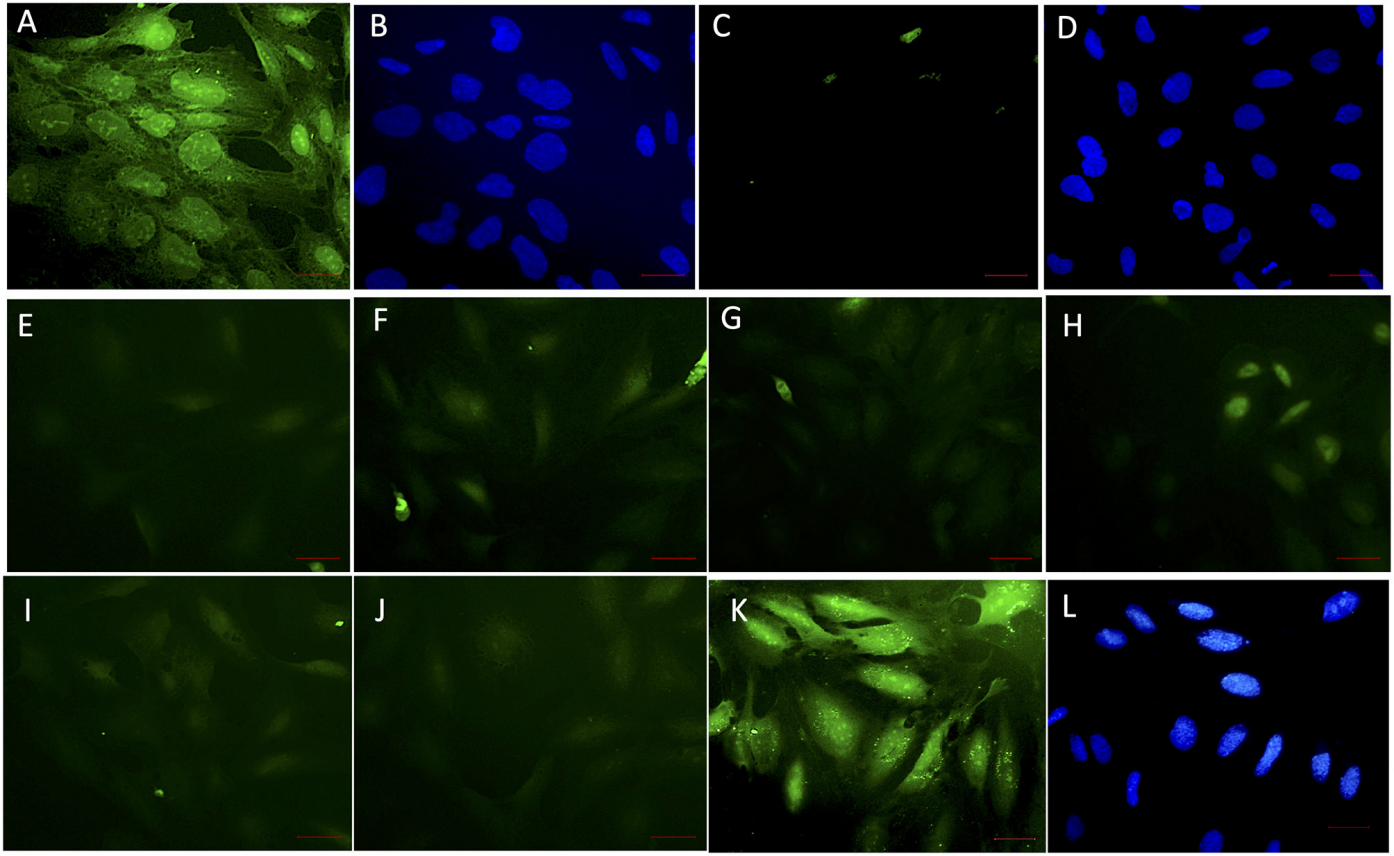
VHH obstructing the interaction of rec-NadA and hBMEC *in situ*. A confluent monolayer of hBMEC incubated with 20 μg of rec-NadA for 1 h [**(A,B)**; positive control]. The interaction was detected with anti-6x His rabbit polyclonal FITC conjugate. Coverslip was mounted with Fluoroshield^Tm^ containing DAPI. Green fluorescence of FITC **(A)**–positive signal and blue fluorescence of DAPI staining nuclei of hBMEC **(B)** confirm adherence of rec-NadA on hBMECs. When the assay was performed with incubation of VHH_B5+F3+G9_, green fluorescence was absent **(C)** but the nuclei of hBMECs were visible with DAPI **(D)**, confirming absence on non-specific fluorescence. Twenty microgram of rec-NadA was preincubated with either VHH_B5_ and VHH_G9_ [20 μg **(E)**, 40 μg **(F)**, and 60 μg **(G)**] or VHH_F3_ and VHH_G9_ at different concentrations [20 μg **(H)**, 40 μg **(I)**, and 60 μg **(J)**] and the premix was added on hBMEC monolayer for 1 h. Likewise, 20 μg of rec-NadA preincubated with 20 μg of non-related VHH [FITC **(K)** and DAPI **(L)**] was added on hBMECs and anti-6x His rabbit polyclonal FITC conjugate was used to detect the interaction. Obsolete FITC signals (green fluorescence) in **(E–J)** indicate reduced adherence of pre-blocked rec-NadA on hBMECs. Whereas, prominent green fluorescence in K confirms that the non-related VHH did not block adherence of rec-NadA on hBMECs. Scale bar (red line)–100 μm.

The ability of soluble VHH in limiting the interaction of rec-NadA with hBMECs was assessed using VHH_**B5**_, VHH_**F3**_, and VHH_**G9**_. rec-NadA pretreated with varying concentrations of VHH (as depicted in Figure 4) was incubated with the monolayer of hBMECs and after stringent washings, bound rec-NadA was detected with anti-His FITC conjugate. Pretreatment caused a noticeable reduction in the rec-NadA-hBMECs interaction (Figures 4E–J) as compared to the cells incubated with unblocked rec-Nada (positive control, Figure 4A). On the other hand, hBMECs incubated with rec-NadA pretreated with non-related VHH showed no compromise in rec-NadA-hBMECs interaction (Figures 4K,L). Whereas, the assay devoid of rec-NadA showed no signals of green fluorescence (negative control, Figure 4C). The specificity of the assay was confirmed by strong FITC fluorescence in positive control and absence of fluorescence in the negative control.

### Large-Scale Production of VHH

**Figure 5:**
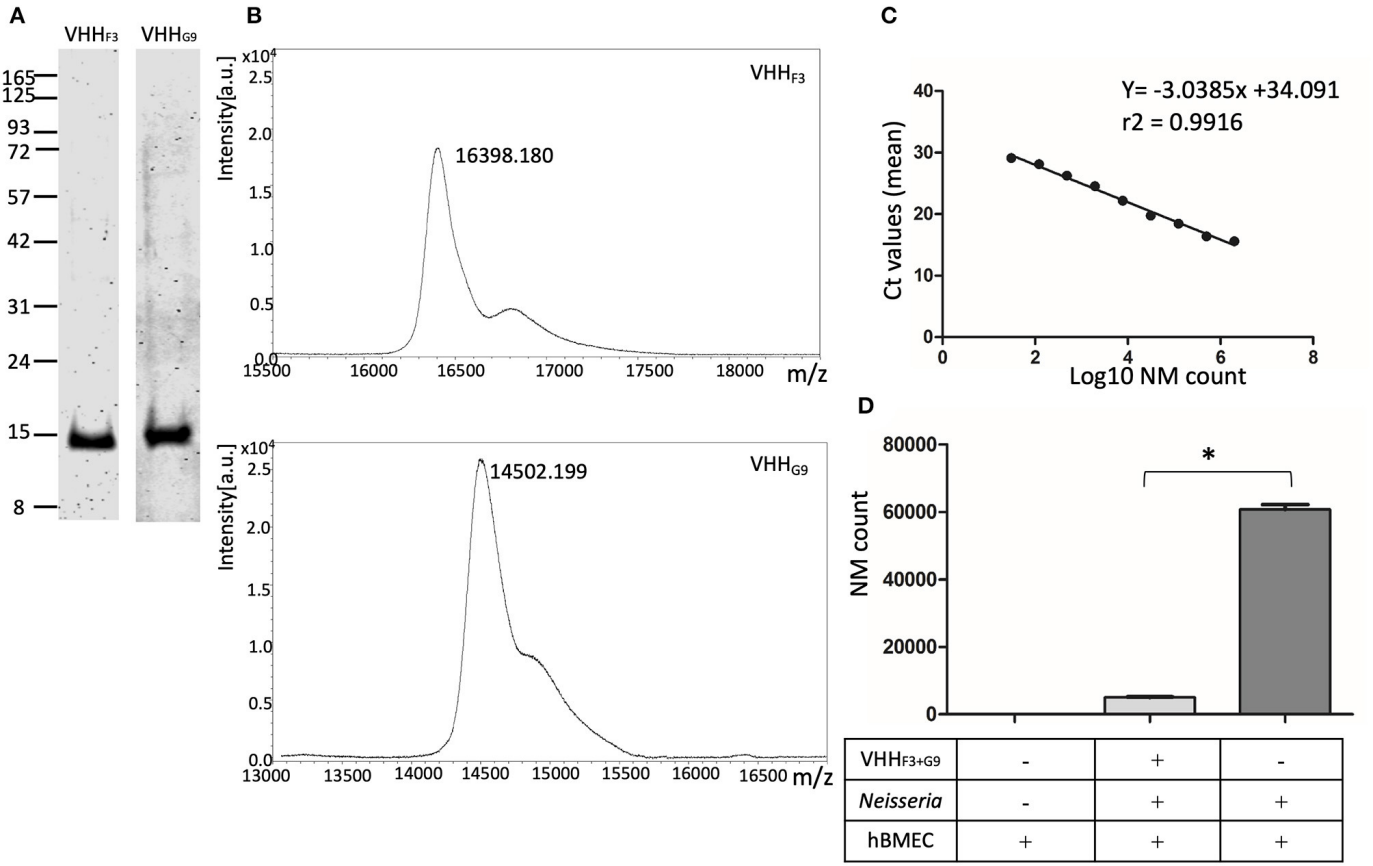
Purified soluble VHHs and their potential to block the adhesion of NM to hBMECs. **(A,B)**–VHH_F3_ and VHH_G9_ were purified by nickel affinity chromatography followed by cation exchange chromatography. SDS PAGE **(A)** and MALDI-MS analysis **(B)** on purified VHH_F3_ and VHH_G9_ are shown. **(C)**–Absolute quantification of NM exposed to hBMECs was performed using qPCR (based on DNA copy numbers) targeting the NadA gene. Two million to 30 bacteria (in 4-fold dilution series) were spiked in hBMEC wells. Fifty nanograms of DNA extracted from the spiked hBMECs was used as a template to quantify DNA copy numbers of NM. Each point in the graph represents mean Ct values. *r*^2−^ Correlation coefficient, slope −3.0385, Y-intercept on x-axis. **(D)**–Adhesion of the number of NMs on hBMECs. NM with (test) or without (positive control) pre-blocking with a combination of VHH_F3_ and VHH_G9_ were incubated with hBMECs. The number of Adherent NM were calculated with qPCR as presented in **(B)**. Statistical significance within the test and positive control group is designated by the asterisk.

The purity of overexpressed VHH_F3_ and VHH_G9_ was high when assessed with SDS-PAGE (Figure 5A) The expected molecular mass of VHH_F3_ (16.07 kDa) and VHH_G9_ (14.18 kDa) coincides with the observed mass of VHH_F3_ (16.39 kDa) and VHH_G9_ (14.5 kDa) on MALDI –MS (Figure 5B). However, VHH_B5_ was poorly overexpressed and showed a high tendency of precipitation during chromatography (data not shown). Thus, VHH_B5_ was omitted from further experiments.

### VHH_F3_ and VHH_G9_ Can Block the Adhesion of NM to hBMECs

Reduction in the number of adhered NM on hBMECs was assessed with quantification of DNA copy numbers. To plot the standard curve, NM at various concentrations (2 × 10^6^ to 30 bacteria, as shown in Supplementary Table 2) were spiked on ~ 2.5 × 10^5^ hBMECs in 6 well–plates. Fifty ng of DNA extracted from each spiked well was quantified for copy numbers of NM targeting NadA gene in qPCR. The mean Ct values ranged from 15.6 (2 × 10^6^ bacteria) to 29.1 (30 bacteria) as presented in Supplementary Table 2. A Linear relationship was observed between the mean Ct values and log_10_ of spiked NM count with a correlation coefficient (*r*^2^) 0.9916, slope (M) −3.0385, and Y-intercept on X-axis (C) 34.091 (Figure 5C).

A significant reduction (*p* < 0.05) in the adhesion of NM to hBMECs due to pretreatment with VHH_F3_ and VHH_G9_ is observed. In the case of pretreated NMs (1 × 10^6^ bacteria pretreated with a combination of VHH_F3_ and VHH_G9_−160 pM each, and then incubated with ~ 2.5 × 10^5^ hBMECs) the mean Ct value was 22.84, which corresponds to 5,013 adherent bacteria. Whereas, in the case of untreated NM (positive control) a mean Ct value was 19.55 that corresponds to 60,702 adherent bacteria (Figure 5D).

### VHH_F3_ and VHH_G9_ Abate Crossing of NM Across *in-vitro* BBB Model

**Figure 6:**
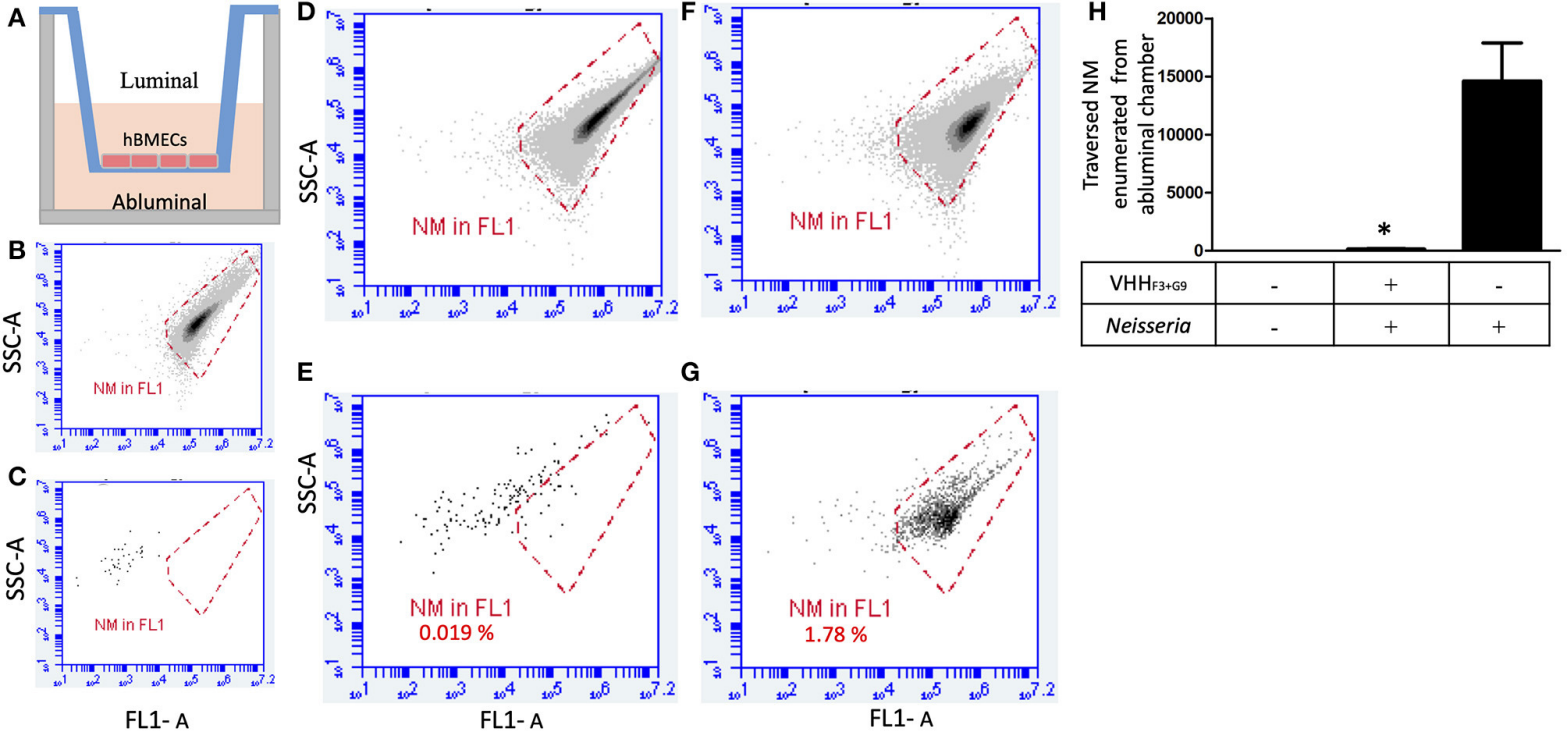
A combination of VHH_F3_ and VHH_G9_ interferes traversal of NM across the BBB model. **(A)**– *In-vitro* BBB model in Transwell consisting of luminal and abluminal chambers separated by a layer of hBMECs (~150,000 cells). **(B)**–1 million NM stained with acridine orange used to plot a gate (red plot). **(C)**–absence of events in DIII medium of abluminal chamber collected from non-seeded BBB model (negative control). One million of pre-blocked NM (with a combination of VHH_F3_ and VHH_G9_) were seeded in the luminal chamber of the BBB model (1 h incubation; MOI = 6). After 1 h incubation, twenty microliters from luminal **(D)** and ab-luminal **(E)** contents were analyzed by flow cytometer. **(F,G)**–Assay was performed in parallel as shown in **(D,E)**, however without pre-blocking of NM. Percentage of NM traversed from luminal chambers to abluminal chambers are mentioned in **(E,G)**. All assays were performed in triplicate and **(D–G)** depicts results of one of the replicates. **(H)**–Bargraph represents the average NM count in abluminal chambers (from triplicates) traversed from luminal chamber. Statistical significance is designated by the asterisk.

One million NM without any treatment (positive control) or preincubated with the combination of VHH_**F3**_ and VHH_**G9**_ (160 pM each) were seeded in the luminal chambers of *in-vitro* BBB model (Figures 6A,D,F). After incubation, average number of NM that traversed to abluminal chambers in positive control was 14,616, whereas in case of preincubated NM the average count of bacteria traversed in abluminal chamber was 142 only (Figures 6A,E,G). Results indicate that the traversal of NM through the BBB model was significantly hindered (*p* < 0.05) due to the pretreatment of bacteria with VHH_**F3**_ and VHH_**G9**_ (Figure 6G).

## Discussion

NM crosses the mucosal barrier of the nasopharyngeal region, survive and replicate in blood and crosses the BBB to invade the meninges. During the invasion, NM is known to express several virulence factors to overwhelm host immune effectors and interact with the components of BBB (Coureuil et al., 2012). Adhesion of NM to BMECs is seen as an obligatory step of meningococcal pathogenesis (Mairey et al., 2006), which promotes the formation of cortical plaques (microvilli like protrusions) surrounding the bacterial colonies (Eugène et al., 2002). Adhesins like outer membrane opacity proteins–Opa and Opc modulate intimate binding of bacteria to hBMECs (Muenzner et al., 2000; Unkmeir et al., 2002), while other adhesins *viz*, auto transports–NhhA, App, MspA, oligomeric coiled-coil adhesin–NadA, β-barrel protein–NspA, two partner secretions–tpsA and tpsB, play important role in NM internalization and pathogenesis (Serruto et al., 2003; Vandeputte-Rutten et al., 2003; Capecchi et al., 2004; Scarselli et al., 2006; Turner et al., 2006; Schmitt et al., 2007). Binding of NadA to hBMECs, epithelial cells, monocytes, macrophages, and monocyte-derived dendritic cells is shown previously (Capecchi et al., 2004; Mazzon et al., 2007; Franzoso et al., 2008; Tavano et al., 2011; Kánová et al., 2018). NM devoid of NadA (knockout mutant) has shown a significant reduction in the adherence of bacteria to human epithelial cells (Capecchi et al., 2004), which highlights its importance in the initial interaction of NM with host cells.

NadA is clustered into two genetically and immunologically distinct groups. The members of a group I (NadA1, NadA2, and NadA3) are associated with pathogenic NM, while members of group II (NadA4, NadA5, and NadA6) are found in carriage strains (Liguori et al., 2018). Recombinant NadA spanning 161 amino acids (S^15^ to V^175^) produced in this study (Figure 1A) shares a 95% sequence identity with the NadA3 variant, of which crystal structure has been characterized recently (Liguori et al., 2018). NadA's globular head domain spans between the amino acid residues A24 to L86 followed by stalk and anchor regions. The head domain of NadA is pivotal for binding the host cell receptors like the human endothelial cell receptor LOX-1 (Scietti et al., 2016). Additionally, it was specified that A33, I38, and Y42 are the key residues of the head domain to interact with LOX-1 (Liguori et al., 2018). These residues were encompassed in the synthetic analog NadA-gd^A33−K69^ (Figure 1), which was shown to bind ~15kDa receptor of hBMEC (Mertinková et al., 2020). Another synthetic analog NadA-cc^L121−K158^, which also takes part in binding the ~15 kDa (Mertinková et al., 2020), lies in the first coiled-coil domain of NadA (Figure 1). This region was also recognized as an adhesion pocket of NadA (Tavano et al., 2011).

It is important to note that, rec-NadA was used to immunize llama in the present study, while synthetic peptides NadA-gd^A33−K69^ and NadA-cc^L121−K158^ were used in panning to select NadA blocking VHHs. Immunization of llama with rec-NadA can elicit antibodies against epitopes that do not overlap the receptor binding sites. For instance, NadA possesses at least 4 antigenic determinants spanning V36–G50, I60–A74, K69–F81, and D75–L93 (Malito et al., 2014). Furthermore, 10 linear and 4 discontinuous epitopes are predicted using homology modeling (Shahsavani et al., 2018). It is reasonable to predict that VHH against several off-target epitopes of NadA might be elicited by immunization. Thus, panning of phages against NadA-gd^A33−K69^ or NadA-cc^L121−K158^ was used to isolate target specific VHH-phage clones. Similar peptide-based panning strategy has been used to isolate VHH against breast carcinoma proteins–Muc-1 (Rahbarizadeh et al., 2004), CD44 (Kavousipour et al., 2018), and ScFv against human angiotensin-I (Cobaugh et al., 2008) and CXC chemokine receptor-2 (Boshuizen et al., 2014). Besides, it was reported that the homogenization of protein with adjuvants may denature some protein molecules before immunization (Friguet et al., 1984), and the elicited antibody pool may contain, target as well as off-target (against unfolded or degraded antigens) antibodies. As we intend to generate VHH to block the interaction between NadA and its receptor on hBMEC, it was obligatory to use synthetic analogs in panning and circumvent VHH generated against denaturated rec-NadA.

In the present study, 20 VHH clones showed stronger binding affinity to the synthetic analogs as compared to the rest of the clones (Figures 3A,B). The same clones were evaluated for their ability to hinder NadA-hBMEC interaction. Interestingly, blocking a single binding site of rec-NadA, i.e., either A33-K69 **(**with NadA-gd^A33−K69^, Figure 3C) or L121-K158 **(**NadA-cc^L121−K158^, Figure 3D) with VHH, did not abrogate the interaction between NadA and 15 kDa receptor of hBMEC. Therefore, we predicted that simultaneously blocking both receptor binding site present on globular domain and coiled-coil region are necessary to hinder interaction between NadA and hBMEC. This hypothesis was also based on the work presented by Tavano and co-workers (Tavano et al., 2011), where only the combination of antibodies raised against NadA peptides spanning the residues 25–39, 94–110, and 109–121 hampered the adhesion of NM to the epithelial cells. Thus, binding pockets present in the globular domain (aa 24 to 87) and adjacent coiled-coil (88 to 133) regions are engaged in cell adhesion (Tavano et al., 2011). Four VHH targeting receptor binding site on globular domain (VHH_A1_, VHH_B5_, VHH_D1_, and VHH_F3_) and three VHH raised against coiled-coil region (VHH_A11_, VHH_E10_, and VHH_G9_) were used in combination to hinder the interaction between NadA and hBMECs. Two combinations, VHH_B5_ + VHH_G9_ and VHH_F3_ + VHH_G9_, could completely block the interaction (Figure 3E). Thus, we interpret that combination of VHH_B5_+VHH_G9_ and VHH_F3_ + VHH_G9_ may have masked both receptor binding sites of NadA, which was not achieved by other VHH combinations. The possible reason being variation in amino acid sequence of CDRs of tested VHHs (Supplementary Figure 6). Especially VHH_G9_ seemed to mask the receptor binding sites of coiled-coil region of NadA completely as opposed to VHH_A11_, VHH_E10._

Hence, the combination of nanobodies, VHH_F3_ (selected against globular domain using NadA-gd^A33−K69^), and VHH_G9_ (selected against CC domain using NadA-cc^L121−K158^), was further tested to block the adhesion of NM to hBMECs and hinder the traversal of NM across *in-vitro* BBB model. A combination of VHH_F3_ and VHH_G9_ significantly reduced the adhesion of NM to the endothelial cells (Figure 5), perhaps due to the complete masking of the receptor binding sites on NadA. Moreover, a significant reduction in the bacterial traversal across the BBB model (Figure 6) suggests the importance of NadA's binding to the endothelial cell receptor. It is well-known that expression of NadA in NM exhibits phase variation due to the presence of tetra nucleotide repeat upstream of *nadA* promoter and negative repressor NadR (Comanducci et al., 2002; Metruccio et al., 2009). The expression levels of NadA are reported to be maximal during the stationary growth phase of NM (Comanducci et al., 2002; Metruccio et al., 2009) Therefore, in the present study NM in their stationary growth phase (overnight grown culture, Tobiason and Seifert, 2010) were used to incubate with VHH_F3_ and VHH_G9._ Similar attempts were made to block NM's another adhesin–opacity protein (OpaJ) with antibodies that hindered its binding with CEACAM1, and non-opsonic interaction of Opa-expressing meningococci with human neutrophils was interrupt (de Jonge et al., 2003). Another study has shown that antisera raised against the peptide GALGQLKVEGAEN of surface-exposed lipoprotein–AniA, inhibited the nitrite reductase activity of *N. gonorrhoeae* which is essential for bacterial colonization and disease development (Shewell et al., 2017). VHH_F3_ and VHH_G9_ were able to block the receptor binding sites of NadA as well as reduced adhesion of NM strain MC58 on hBMECs and its traversal across BBB *in vitro*, however, it is necessary to evaluate blocking ability of VHHs against other NM strains to testify general utility of the produced nanobodies.

Phage display, employed in the study to select VHH, is one of the robust display techniques often used to generate diversified antibody fragments. Classical phage display includes antigen immobilization, incubation of antibody-phage library with antigen, extensive washings, elution of bound phages, and amplification of eluted phages in *E. coli* (with or without superinfection of helper phages) (Shim, 2017). The entire process is repeated three to four times (rounds of panning) to enrich phages with the highest affinity to the antigen. Antibodies fused to PIII coat protein of M13 bacteriophage is a common strategy used to display the antibody on phage particle, while the phagemid and helper phage systems are employed for phage packaging (Barbas et al., 1991; Breitling et al., 1991; Hoogenboom et al., 1991; Marks et al., 1991). Helper phage with wild-type pIII gene, like M13KO7, can produce only a small percentage of the total phage population that carries an antibody fragment (Carmen and Jermutus, 2002). This is mainly due to the usage of phagemids encoding the antibody–pIII fusion protein requiring a helper phage carrying a predominant wild-type pIII gene to supply other proteins for the phage assembly. This drawback compromises the efficiency of the panning steps and antibody selection. Of late, hyper phage M13 K07ΔpIII was introduced whose pIII gene was deleted (leaving the promoter and signal peptide DNA intact) from the genome yet phenotypically it expressed wild type PIII (Rondot et al., 2001). As a result, the efficiency of the antibody display and antigen-binding activity was increased to ~ 400-fold (Rondot et al., 2001). In the present study, phagemid carrying only CT of PIII was used to fuse with VHH, while M13 K07ΔpIII was used to packaging the phages. Thus, the packaged phage offspring were displaying VHH on the CT of PIII and were noninfectious due to the absence of N1 and N2 domains (Supplementary Figure 5), which allowed us to perform one round of panning.

A single round of panning was shown to be sufficient to obtain ligands (binders) for anti-colorectal cancer cells (Williams and Sharon, 2002), polyclonal antibodies against surface glycoproteins of protozoan parasite–*Cryptosporidium parvum* (Chen et al., 2003), substrates for matrix metalloprotease-9 (Kridel et al., 2001) and binders of pluripotent stem cells (Derda et al., 2010). Derda and their co-workers observed that the diversity of phage-library displaying peptides decreased abruptly as the rounds increased (Derda et al., 2010). Their finding is considered as a major pitfall of phage amplification in multiple rounds of panning that incorporates two different selection pressure on phages viz, binding affinity, and the difference in growth rate (Derda et al., 2011). NGS based sequencing of eluted phages after each round of panning has proved enrichment of non-specific binders ('T Hoen et al., 2012). Since single panning preserves a broader repertoire of phages, we followed the same to attain a diversified library and subsequently increase the chances of retaining blocking nanobodies. In the present study eluted phages were subjected to nucleic acid extraction and amplification of VHH encoding fragment with PCR. Amplicons were cloned into the expression vector to produce soluble VHHs. This phage-DNA-PCR strategy has also been conveniently used to quantify phages specifically bound to vascular endothelium (Ballard et al., 2006), obtain the sequence of αv integrin-binding ligand (Dias-Neto et al., 2009) and identifying epitopes of a prominent peanut allergen (Christiansen et al., 2015).

VHH sequence typically has two cysteine residues one in framework region 1 and another in framework region 3 (Figure 2A; Supplementary Figure 6) (Mitchell and Colwell, 2018). The formation of disulfide bonds between these two cysteine residues is essential to maintain the proper folding and functionality of the nanobodies (de Marco, 2015). The expression system used in the present study, *E. coli* SHuffle T7 express system, enables the formation of stabilized disulfide bond thanks to its oxidative cytoplasm (Lobstein et al., 2012).

## Conclusion

VHHs against two receptor binding sites of NadA of NM were produced successfully in the present study using a modified phage display approach. A combination of VHH_F3_ targeting NadA-gd^A33−K69^ and VHH_G9_ targeting NadA-cc^L121−K158^ was able to block the interaction between rec-NadA and 15kDa protein of hBMEC. The same combination of VHH was able to interfere with the adhesion of NM to hBMECs and traversal of *Neisseria* through the *in-vitro* BBB model. Further study on the assessment of the therapeutic potential of both VHHs is highly demanding and is the subject of ongoing research.

## Data Availability Statement

The datasets generated for this study can be found in online repositories. The names of the repository/repositories and accession number(s) can be found at: NCBI GenBank [accession: MT637228-MT637230, MW250877-MW250880, and MW286772-MW286786].

## Ethics Statement

The animal study was reviewed and approved by Ethical committee for handling animals of University of veterinary medicine and pharmacy in Kosice, Slovakia, approved according to the regulations of Slovak government number 377/2012.

## Author Contributions

MB conceived the project and designed experiments. Experiments were performed by AK. JČ made immunization. hBMECs culture was carried out by EM. *In-vitro* BBB was set by PMa and sequencing was performed by PMe and KB. AK and MB prepared the manuscript. KB and MB received funding. All authors read and approved the final manuscript.

## Conflict of Interest

The authors declare that the research was conducted in the absence of any commercial or financial relationships that could be construed as a potential conflict of interest.

## Abbreviations

NadA: *Neisseria* adhesin A
hBMEC: human brain microvascular endothelial cells
rec-NadA: recombinant NadA
BBB: blood-brain barrier
NM: *Neisseria meningitides*
VHH: Single variable domain on a heavy chain
GFP: green fluorescent protein
Ct: threshold cycle
PBMC: Peripheral blood mononuclear cells
TTBS: 1xTris buffered saline + Tween 20
BSA: bovine serum albumin
qPCR: quantitative real-time PCR.
