# Supplementary material for: Single Domain Antibodies Targeting Receptor Binding Pockets of NadA Restrain Adhesion of Neisseria meningitidis to Human Brain Microvascular Endothelial Cells
Source: Front Mol Biosci. 2020 Dec 23;7:573281. doi: 10.3389/fmolb.2020.573281 (PMC7785856; doi:10.3389/fmolb.2020.573281)
Supplement: Supplementary file 1 [file Data_Sheet_1.PDF]

Additional File 1

Single domain antibodies targeting receptor binding pockets of NadA restrain adhesion of *Neisseria meningitides* to human brain microvascular endothelial cells

Amod Kulkarni[1,2], Evelína Mochnáčová[1], Petra Majerova[2], Ján Čurlík[1], Katarína Bhide[1], Patrícia Mertinková [1] and Mangesh Bhide[1,2*]

[1]Laboratory of Biomedical Microbiology and Immunology, The University of Veterinary Medicine and Pharmacy, Komenskeho 73, 04001, Kosice, Slovakia,

[2]Institute of Neuroimmunology of Slovak Academy of Sciences, 84510, Bratislava, Slovakia

Supplementary Figures

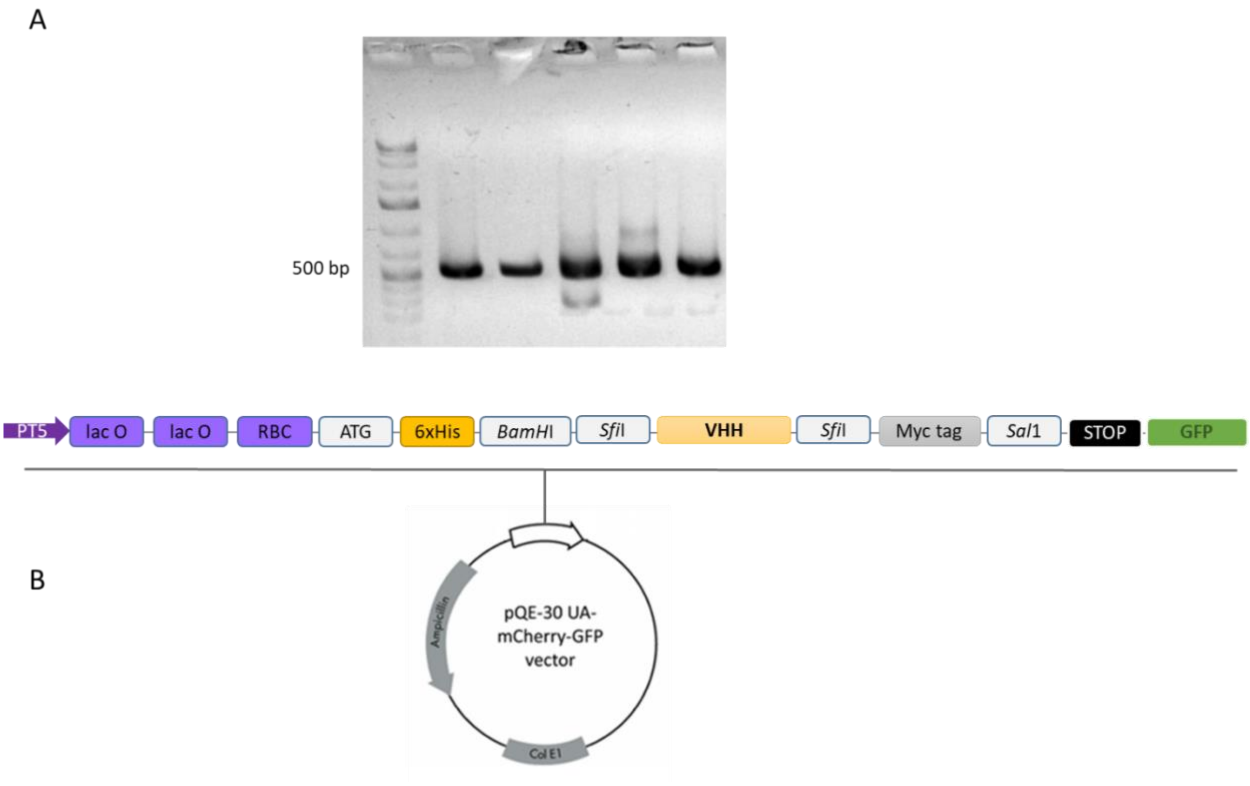

Supplementary Figure 1: Amplification of VHH from panned phages and a vector map of pQE-30-UA-mCherry-Stop-GFP used to insert VHH gene. Panel A - After biopanning of phages with NadA-gd$^{A33-K69}$ and NadA-cc$^{L121-K158}$, gene fragment encoding VHH was amplified from the DNA of eluted phages. The PCR amplified VHH (~500 bp) was excised from gel and purified. Panel B – Purified PCR products were digested with *Sfi*I and ligation into *in-house* modified pQE-30-UA-mCherry-Stop-GFP vector.

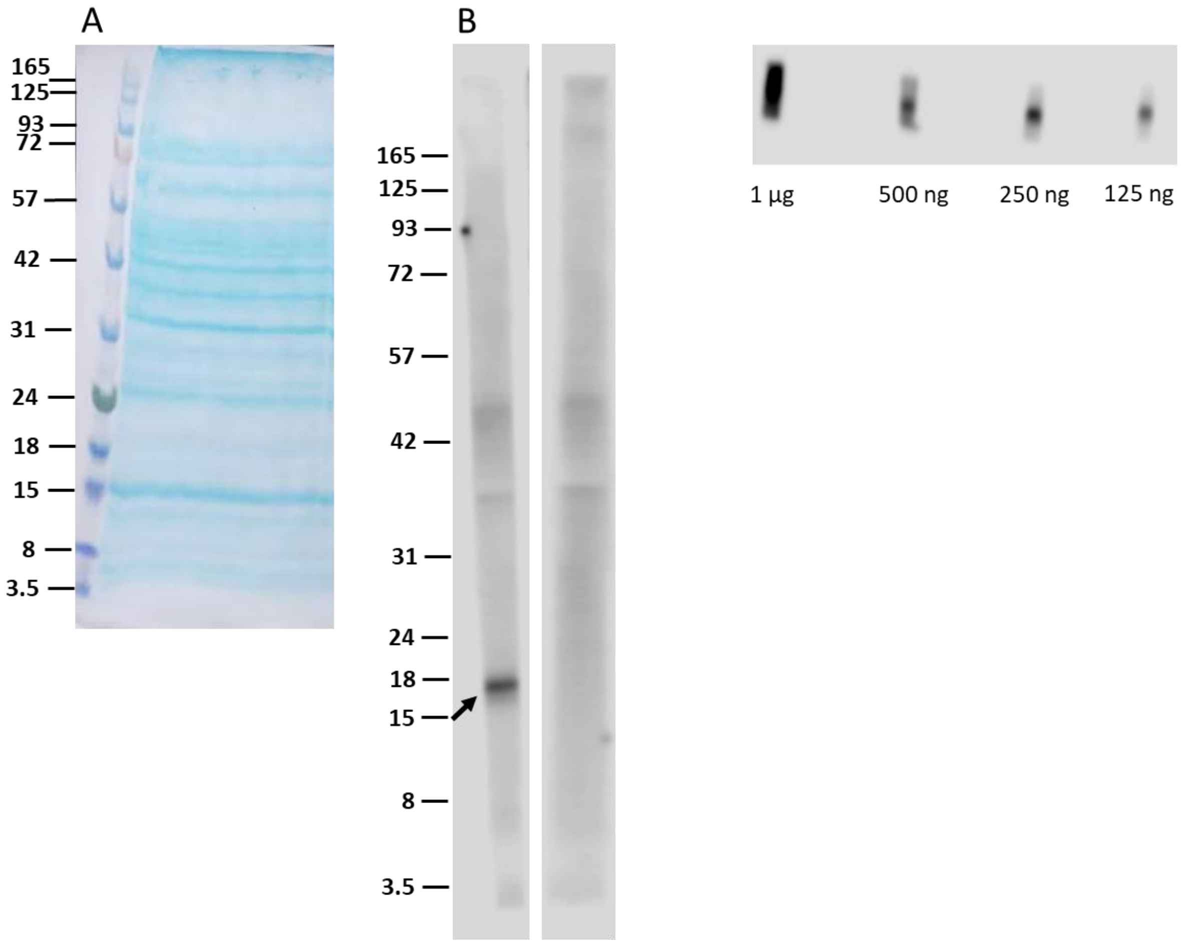

Anti-6x His AbA	+	+
rec-NadA	+	-
hBMEC	+	+

Supplementary Figure 2. Interaction of rec-NadA and hBMEC proteins by western blot. Panel A - Four hundred micrograms of proteins extracted from hBMEC lysate was electroblotted on NC membrane and cut into 5 mm vertical strips. Panel B – Strips were used in western blot to identify the interaction rec-NadA with proteins of hBMECs. Interaction was detected by anti-6x His antibody. Positive reaction of western blot with 15kDa hBMEC protein is marked with arrow. Protein strip incubated with PBS instead of rec-NadA served as negative control. Panel C – various concentrations of rec-NadA was incubated with 15kDa hBMEC protein. Interaction was detected with anti-6x His antibody.

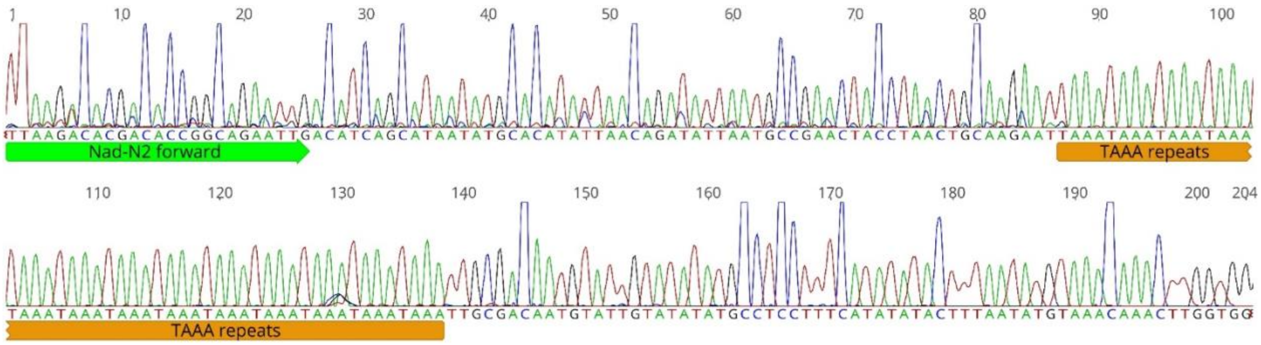

Supplementary Figure 3: Sequence of region upstream of NadA promoter of NM (Strain MC58, isolate - M1/03). Nad N2 primer (Metruccio et al. 2009) was used to sequence the region upstream of NadA promoter from the genomic DNA of NM (Strain MC58, isolate - M1/03) used in the present study. tetranucleotide tract -TAAA repeat region within the sequence is depicted.

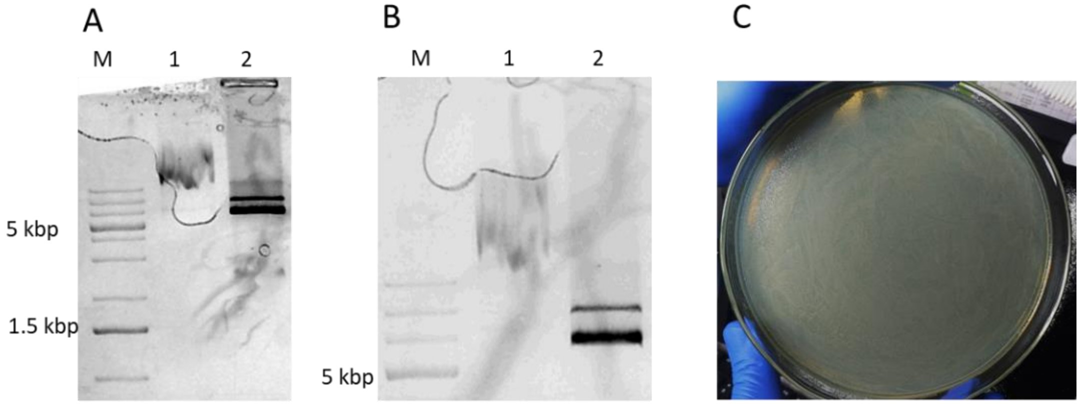

Supplementary Figure 4. **Separation of digested pJB12 phagemid and illustration of VHH-GFP-*E. coli* library**. Panels A and B - pJB12 phagemid was digested with *Sfi*I enzyme and subjected to electrophoresis. Photo documentation was performed after 3h (Panel A) and 5 h (Panel B) of electrophoresis. Lane M: molecular weight marker, Lane 1 (in panels A and B) undigested pJb12 phagemid and Lane 2 (in panels A and B) digested pJb12 phagemid. Panel C - *E. coli* transformants carrying VHH in pJB12 phagemid grown on LB plate (VHH – E. coli library).

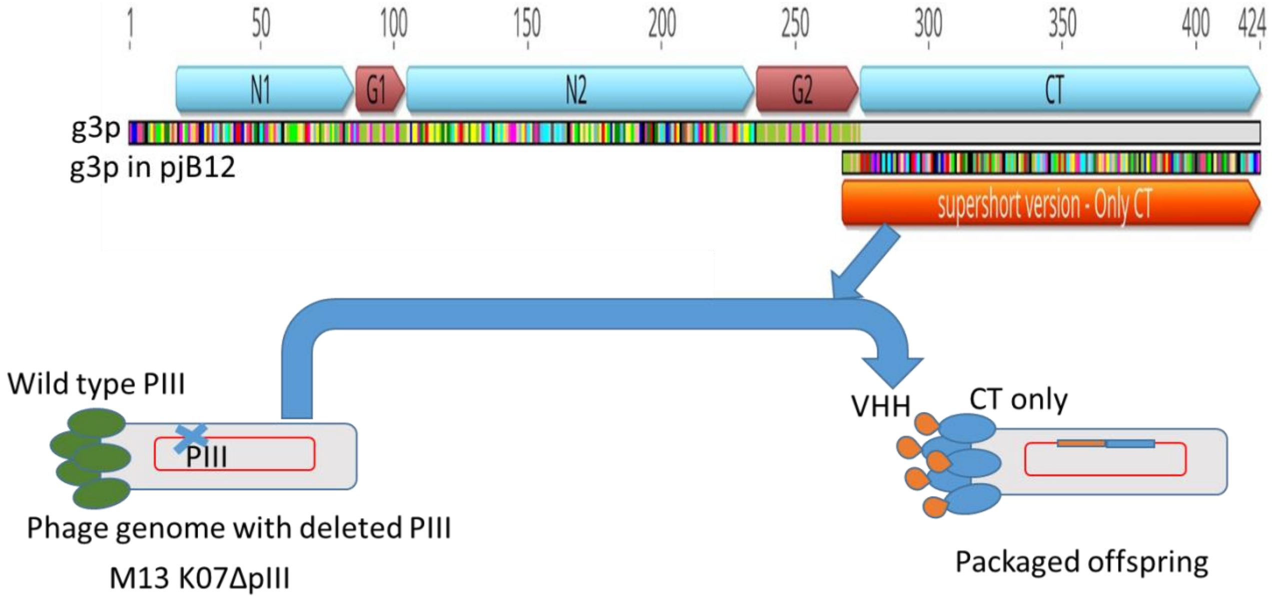

Supplementary figure 5: Structure of g3p (PIII) protein. The N1 domain interacts with the membrane proteins of E. coli -TolA. The N2 domain is obligatory to bind the F pilus and the CT domain takes part in phage assembly and release of phages from E. coli. G1 and G2 shown in the figure are the glycine-rich linkers, which provides flexibility to the adjacent domains during the infection process. In the present study we used super short version of phagemid where in only the CT domain is retained. VHH is cloned to N terminus of the CT. The phage used for packaging is devoid of PIII (only promoter and signal peptide are retained in genome), however phenotypically expressing wild-type PIII (green oval shapes in M13 K07ΔpIII) on its surface. Thus offspring phage can be packaged with PIII with CT only presenting VHH on N-terminus (blue oval shapes with red VHH in offspring). Offspring devoid of N1 and N2 cannot interact with TolA of *E. coli* and bind F pilus, receptively thus are non-infective.

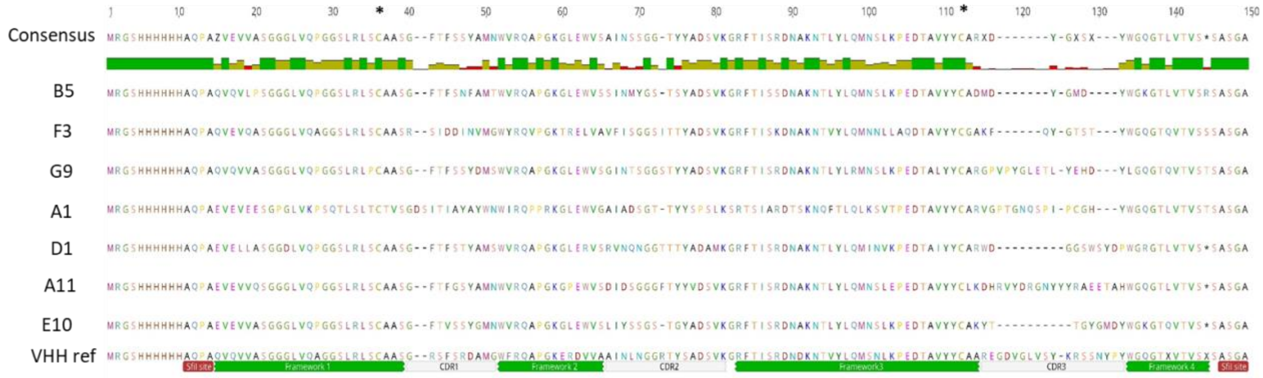

Supplementary Figure 6: Vector map of pQE-30-UA-mCherry-Stop-GFP used to produce soluble VHH and sequences of VHH$_{B5}$, VHH$_{F3}$ and VHH$_{G9}$, VHH$_{A1}$, VHHD1, VHH$_{A11}$, and VHH$_{E10}$. Sequences of VHH$_{B5}$, VHH$_{F3}$ VHH$_{G9}$, VHH$_{A1}$, VHH$_{D1}$, VHH$_{A11}$, and VHH$_{E10}$ are aligned to VHH reference sequence. Framework regions, CDRs and SfiI enzyme sites are marked. Star indicates cysteine residue.

Supplementary Tables

Supplementary Table 1: Sequence of Synthetic analogues of receptor binding sites of NadA

Name	Peptide sequence
NadA-gd$^{A33-K69}$	AATVAIVAAYNNGQEINGFKAGETIYDIGEDGTITQK (Biotin)
NadA-cc$^{L121-K158}$	LADTDAALADTDAALDETTNALNKLGENITTFAEETK (Biotin)

Supplementary Table 2: Number of NM used to enumerate DNA copy numbers in qPCR

Dilutions	NM count	Log10 NM count	Ct values (mean)
0	2000000	6.30103	15.56778
1	500000	5.69897	16.39478
2	125000	5.09691	18.43364
3	31250	4.49485	19.75164
4	7812	3.89279	22.19471
5	1953	3.29073	24.55309
6	488	2.68867	26.23803
7	122	2.08661	28.12713
8	30	1.48455	29.10653
